# Supplementary material for: Reconciling Mining with the Conservation of Cave Biodiversity: A Quantitative Baseline to Help Establish Conservation Priorities
Source: PLoS One. 2016 Dec 20;11(12):e0168348. doi: 10.1371/journal.pone.0168348 (PMC5173368; doi:10.1371/journal.pone.0168348)
Supplement: S1 Dataset — (ZIP) [file pone.0168348.s002.zip › Taxa/Serra Norte/SN_2013/N5S/Lista N5S-30.pdf]

CAVIDADE N5S-0030

Classe	Ordem	Fam/Outros	Gên/Outros	Espécie	S-U	U-S	Zon.
Arachnida	Acari	(Parasitengonina)		sp.1		X	A
Arachnida	Acari	(Parasitengonina)		sp.2	X		P
Arachnida	Acari	(Parasitengonina)		sp.5		X	A
Arachnida	Acari	(Parasitengonina)		sp.7		X	E
Arachnida	Acari	Argasidae	*Ornithodoros*	sp.1	X		P/A
Arachnida	Acari	Ixodidae	*Amblyomma*	sp.1		X	E/A
Arachnida	Acari	Macronyssidae		sp.1		X	A
Arachnida	Acari	Rosesteniidae		sp.1		X	A
Arachnida	Acari			sp.1	X		A
Arachnida	Acari			sp.3	X	X	E/A
Arachnida	Amblypygi	Phrynidae	*Heterophrynus*	*longicornis*	1	5	E/P/A
Arachnida	Araneae	Caponiidae		sp.1	X	X	P/A
Arachnida	Araneae	Drymusidae	*Drymusa*	*spelunca*	X	X	E
Arachnida	Araneae	Ochyroceratidae	*Ochyrocera*	sp.1		X	E
Arachnida	Araneae	Ochyroceratidae	*Ochyrocera*	sp.3	X	X	A
Arachnida	Araneae	Pholcidae	*Mesabolivar*	*eberhard*		X	E
Arachnida	Araneae	Pholcidae	*Mesabolivar*	sp.1		X	E
Arachnida	Araneae	Pholcidae		jovem	X	X	A
Arachnida	Araneae	Salticidae		sp.2		X	E
Arachnida	Araneae	Salticidae		sp.6		X	E
Arachnida	Araneae	Theraphosidae		jovem		1	E
Arachnida	Araneae	Theridiidae	*Achaearanea*	sp.1		X	E
Arachnida	Araneae	Theridiosomatidae	*Plato*	sp.1	X	X	E/P/A
Arachnida	Opiliones	Escadabiidae		sp.1	X	X	P/A
Arachnida	Opiliones	Escadabiidae		sp.2	X		P
Arachnida	Opiliones	Sclerosomatidae	*Prionostema*	sp.1		X	E
Arachnida	Opiliones	Stygnidae		jovem	1		E
Arachnida	Palpigradi	Eukoeneniidae	*Allokoenenia*	sp.1	X		P/A
Arachnida	Palpigradi	Eukoeneniidae	*Eukoenenia*	sp.1	X		E
Arachnida	Palpigradi	Eukoeneniidae	*Leptokoenenia*	*thalassophobica*	X		A
Arachnida	Pseudoscorpiones	Chernetidae	*Spelaeochernes*	sp.1	X	X	P/A
Arachnida	Pseudoscorpiones	Chthoniidae	*Pseudochthonius*	sp.1		X	E/A
Arachnida	Pseudoscorpiones	Chthoniidae	*Pseudochthonius*	sp.2	X		A
Arachnida	Pseudoscorpiones	Chthoniidae		jovem	X		A
Arachnida	Pseudoscorpiones	Chthoniidae		sp.2		X	A
Chilopoda	Scolopendromorpha	Scolopocryptopidae	*Newportia*	sp.1	1	1	E
Diplopoda	Polydesmida	Fuhrmannodesmidae		sp.1	X		A
Diplopoda	Polydesmida	Pyrgodesmidae		sp.1	X	X	P/A
Diplopoda	Spirostreptida	Pseudonannolenidae	*Pseudonannolene*	sp.1	2	4	E/P/A
Entognatha	Collembola	Isotomidae		sp.1	X	X	P/A
Entognatha	Collembola	Paronellidae		sp.1	X		P
Entognatha	Diplura	Japygidae		sp.1	X		E/P
Insecta	Blattodea	Blaberidae		sp.2	6		A
Insecta	Blattodea	Polyphagidae		sp.1	4	5	P
Insecta	Coleoptera	Aderidae		sp.2		X	A
Insecta	Coleoptera	Carabidae	*Coarazuphium*	sp.n.1	X	X	A
Insecta	Coleoptera	Elateridae		sp.1	X	X	A
Insecta	Coleoptera	Tenebrionidae		jovem	X	X	A
Insecta	Diptera	Cecidomyiidae	Cecidomyiinae	sp.	X		P

Insecta	Diptera	Ceratopogonidae		sp.	X	X	P
Insecta	Diptera	Culicidae	*Culex*	sp.		X	E
Insecta	Diptera	Ephydridae		sp.	X		A
Insecta	Diptera	Phoridae	Phorinae	sp.	X		A
Insecta	Diptera	Psychodidae	*Edentomyia*	*piauiensis*	X	X	P
Insecta	Diptera	Psychodidae	*Evandromyia*	*saulensis*	X		E
Insecta	Diptera	Psychodidae	*Pintomyia*	*gruta*	X		A
Insecta	Diptera	Psychodidae	*Sciopemyia*	*sordellii*		X	E
Insecta	Diptera	Streblidae	*Trichobius*	sp.		X	A
Insecta	Diptera	Tipulidae	Tipulinae	sp.	X		P
Insecta	Heteroptera	Ceratocombidae		sp.1	X		P
Insecta	Heteroptera	Reduviidae	Emesinae	jovem	1		A
Insecta	Heteroptera	Reduviidae	*Panstrongylus*	sp.	1		A
Insecta	Heteroptera	Reduviidae	Triatominae	jovem	3	4	A
Insecta	Hymenoptera	Formicidae	*Camponotus*	sp.1	X	X	E/P/A
Insecta	Hymenoptera	Formicidae	*Crematogaster*	sp.1	X		P
Insecta	Isoptera	Termitidae	*Cortaritermes*	*silvestrii*		X	E/P/A
Insecta	Lepidoptera	Tineoidea		sp.3		X	A
Insecta	Lepidoptera	Tineoidea		sp.5		X	P/A
Insecta	Lepidoptera			jovem	X	X	P/A
Insecta	Orthoptera	Phalangopsidae	*Phalangopsis*	sp.	150	250	P/A
Insecta	Psocoptera	Ptiloneuridae	*Ptiloneura*	sp.1		X	E
Insecta	Thysanura	Ateluridae		sp.1	X		P
Malacostraca	Isopoda	Scleropactidae	*Circoniscus*	*buckupi*	X	X	A
Mammalia	Chiroptera	Emballonuridae	*Peropteryx*	sp.		10	E/P
Mammalia	Chiroptera	Furipteridae	*Furipterus*	*horrens*		150	P/A
Mammalia	Chiroptera	Phyllostomidae	Glossophaginae	sp.		50	P/A
Mammalia	Rodentia	Cricetidae	*Rhipidomys*	sp.		1	P
